# Study on Filter Cake Removal Fluid of EZFLOW Weak Gel Drilling Fluid

**DOI:** 10.3390/gels11050347

**Published:** 2025-05-08

**Authors:** Haohan Hu, Youlin Hu, Xuejing Weng

**Affiliations:** 1 College of Petroleum Engineering, Yangtze University, Wuhan 430100, China; huhaohan2005@126.com; 2 State Key Laboratory of Petroleum Resources and Prospecting, China University of Petroleum at Beijing, Beijing 102249, China; 2022210224@student.cup.edu.cn

**Keywords:** weak gel drilling fluid, performance mechanism, filter cake removal, reservoir protection

## Abstract

EZFLOW weak gel drilling fluid, a drilling fluid system with distinctive internal architecture, has been extensively implemented in horizontal well drilling operations at the Western South China Sea oilfields. Its unique internal structure causes specific functional mechanisms. The rheological mechanism was investigated through microstructural characterization, revealing that the microstructure comprises a reversible network structure with sol particles either encapsulated within the network or embedded at nodal points. This distinctive spatial network configuration endows the system with exceptional rheological properties. The plugging mechanism was elucidated via pre- and post-PPA test characterization of sand disc surface morphology. Experimental results demonstrate that the rheology modifier EZVIS forms deformable aggregates and films through intermolecular or intramolecular association in aqueous solutions, effectively plugging micro-nano pores/throats and microfractures to inhibit drilling fluid filtrate invasion. Concurrently, the rigid plugging material EZCARB establishes physical barriers at micro-nano pores/throats through bridging mechanisms. Notably, the dense filter cake formed by EZFLOW weak gel drilling fluid exhibits poor flowback characteristics, potentially inducing reservoir damage. Based on mechanistic analyses of rheological behavior, plugging performance, and filter cake composition, a filter cake removal fluid formulation was developed through: (1) creation of retarded acid HWCP to degrade polymer EZVIS and dissolve temporary plugging agent EZCARB; (2) development of corrosion inhibitor HWCI to mitigate corrosion rates. Laboratory evaluations demonstrated effective filter cake elimination and reservoir protection capabilities. Post-treatment analysis of EZFLOW-contaminated reservoir cores showed complete filter cake removal at core end faces with permeability recovery values exceeding 95%, indicating superior filter cake dissolution capacity and reservoir protection performance that significantly reduces formation damage.

## 1. Introduction

The weak gel drilling fluid is a novel drilling fluid system with a special internal structure; it exhibits superior properties such as high yield point to plastic viscosity ratio, elevated low shear rate viscosity (LSRV > 22,000 mPa·s), time-independent gel strength, and excellent wellbore cleaning capacity [1,2,3,4,5,6,7,8,9,10]. The distinctive internal architecture of weak gel drilling fluids fundamentally differentiates their functional mechanisms from conventional polymer-based systems. In addition, compared to common polymer sulfonate drilling fluids, the environmental performance is better [11,12,13,14,15,16,17,18,19,20,21]. Li et al. [22] employed free-radical polymerization to synthesize a weak gel water-based drilling fluid gel former (WGA) using the monomers acrylamide (AM), 2-acrylamido-2-methylpropane sulfonic acid (AMPS), and 2-acrylamidoalkylsulfonic acid. Under hydrophobic forces, hydrogen bonding of polymer molecules, and intermolecular Coulomb forces, the aqueous solution of WGA had a 3D network structure with a completely extended conformation. The network structure of the gel agent was formed by the combination of the electrostatic and hydrophobic forces and hydrogen bonding. As the concentration of the gel agent increased, the molecular chain association was enhanced, and the network structure became denser. Shanmugam et al. [23] explored the use of two novel ultra-high molecular weight branched block copolymer (UHMW-BCP) microgels that were able to tolerate different water-based drilling muds, including freshwater, potassium chloride brine, and lime muds. Li et al. [24] studied the internal network structure of the weak-gel-type clay-free and water-based drilling fluid under different temperatures and salt contents through environmental scanning electron microscopy, and the results showed that its microstructure comprises a three-dimensional network, in which sol particles are wrapped or embedded at the network structure nodes. Its network density and skeleton became thinner as temperature increased, whereas they became coarse and loose as the salt content increased. Huang et al. [25] introduced the non-clay weak gel drilling fluid system; the drilling fluid system had excellent temperature-resisting and salt-resisting characteristics, a strong ability to suspend debris, and strong inhibitory action. Its rheological property could meet the needs of the long horizontal section of drilling engineering and the reservoir protection effect was excellent. Luo et al. [26] engineered a solid-free weak gel drilling fluid where polymer PF-VI (guaragum), under the action of crosslinker JLJ (borax), formed a cross-linked network in aqueous solutions to enhance viscosity and gel strength. Wang et al. [27] developed a set of HRD weak gel drilling fluid suitable for horizontal well drilling based on the synergistic effect of different types of polymers through interaction. The exceptional network structure of these systems endows the drilling fluid with unique structural properties. Shi et al. [28] characterized the dynamic retention process of GG, HPG, and CMG in sandstone cores; the aggregation and entanglement characteristics of the three types of guar gum in sandstone were analyzed from the structure of guar gum. Wu et al. [29] compared the viscosity, molecular weight, and particle size of the fracturing fluid after gel breakage prepared by GG and HPAM as viscosifiers, as well as evaluating their damage to the core. Huang et al. [30] constructed a supramolecular reinforced gel (SRG) fracturing fluid by strengthening the supramolecular force between polymers in order to reduce reservoir permeability damage caused by incomplete gel breaking. In order to improve the rheological properties and reduce the filtration properties of the drilling fluid used for drilling the oil-bearing zone horizontally, Neamat et al. [31] modified and optimized the reservoir drill-in fluid (RDF). Hamid et al. [32] investigated the performance of hybrid gels enhanced with nanoparticles (NPs) for controlling fluid loss during drilling operations. The weak gel drilling fluid has excellent cuttings carrying ability. It meets the requirements for drilling engineering and environmental protection. It is low in apparent viscosity, and gel strength slightly varies with time. It also has better thixotropy, and it quickly forms a gel during dilution shearing. All properties mentioned above satisfy the requirements for drilling a horizontal well. However, the dense filter cake formed by the weak gel drilling fluid impedes post-production flowback, as it can damage the reservoir. In addition, adsorption of the polymer in the weak gel drilling fluid on the pore skeleton particles of the reservoir blocks oil and gas seepage channels and reduces permeability and well productivity. Li et al. [33] analyzed the microscopic damage mechanisms of the gel fluids by thin section petrography, X-ray diffraction, rate-controlled mercury intrusion, scanning electron microscopy, and wettability tests.

The EZFLOW weak gel drilling fluid has excellent cuttings carrying ability. It is biodegradable and innocuous, which meets the requirements for drilling engineering and environmental protection. It provides excellent salt tolerance and good temperature resistance. All properties mentioned above satisfy the requirements for drilling a horizontal well. It has been widely adopted in horizontal well drilling operations in the Western South China Sea oilfields. The microstructure of the EZFLOW weak gel drilling fluid was examined using scanning electron microscopy (SEM), and the correlation between its internal network state and rheological behavior and plugging performance was analyzed in detail. The mechanism of rheology and plugging performance of EZFLOW weak gel drilling fluid was studied by analyzing the microstructure of drilling fluid and the characterization of surface morphology of sand tray before and after PPA experiment.

The dense filter cake formed by EZFLOW weak gel drilling fluid impedes post-production flowback, causing reservoir damage. The adsorption of the polymer EZVIS in the EZFLOW weak gel drilling fluid on the pore skeleton particles of the reservoir reduces permeability. Taghdimi et al. [34] comprehensively analyzed polymer induced reservoir damage mechanisms using scanning electron microscopy (SEM), energy-dispersive X-ray spectroscopy (EDS), gas chromatography (GC), and mass spectrometry (MS), providing a theoretical foundation for gel-breaking and damage mitigation. The invasion of the acid-soluble bridging material EZCARB in the EZFLOW weak gel drilling fluid into formation pores blocks oil and gas seepage channels. Therefore, in order to effectively protect the reservoir, it is necessary to use filter cake eliminating fluid to relieve the damage of filter cake, polymer EZVIS, and acid soluble temporary plugging material EZCARB to the reservoir, and to restore the oil and gas seepage channel. Current industry practices predominantly rely on strong acids, potent oxidizers, and bioenzymes to degrade polymeric components in filter cakes and near-wellbore regions. However, strong acids exhibit rapid reaction rates, leading to incomplete filter cake removal and mud cake breakthrough. Oxidizers face limitations due to their reactivity, secondary contamination risks, and operational hazards. Bioenzymes suffer from poor compatibility and sensitivity to temperature and pH [35,36].

To address these challenges, a filter cake removal fluid was developed based on the composition of EZFLOW filter cake. Using filtered seawater as the base fluid, a retarded acid (HWCP) was employed to degrade residual polymers in pores/fractures and dissolve EZCARB within the filter cake and invaded zones. A corrosion inhibitor (HWCI) was incorporated to mitigate corrosion rates. Optimal HWCP concentration (6%) and HWCI dosage (3%) were determined through evaluations of polymer degradation efficiency (for 0.8% EZVIS seawater solutions), EZCARB dissolution rates, and reservoir cuttings dissolution, combined with static coupon weight loss tests. The formulated system comprises filtered seawater + 6% HWCP + 3% HWCI. Core immersion tests confirmed thorough filter cake removal at core end faces. Core flow experiments and SEM analyses demonstrated effective reservoir protection, significantly reducing formation damage. Field applications in three wells in the Western South China Sea validated the system’s superior plugging removal and production enhancement capabilities.

## 2. Results and Discussion

### 2.1. Mechanism of Weak Gel Drilling Fluid

The EZFLOW weak gel drilling fluid used in horizontal wells at the Western South China Sea oilfields is formulated as follows: seawater + 0.2% Na_2_CO_3_ + 0.2% NaOH + 0.8% EZVIS + 1.5% EZFLO + 8% EZCARB + 3% polyalcohol JLX. Potassium formate is used to adjust the drilling fluid density.

#### 2.1.1. Rheological Properties

**Figure 1:**
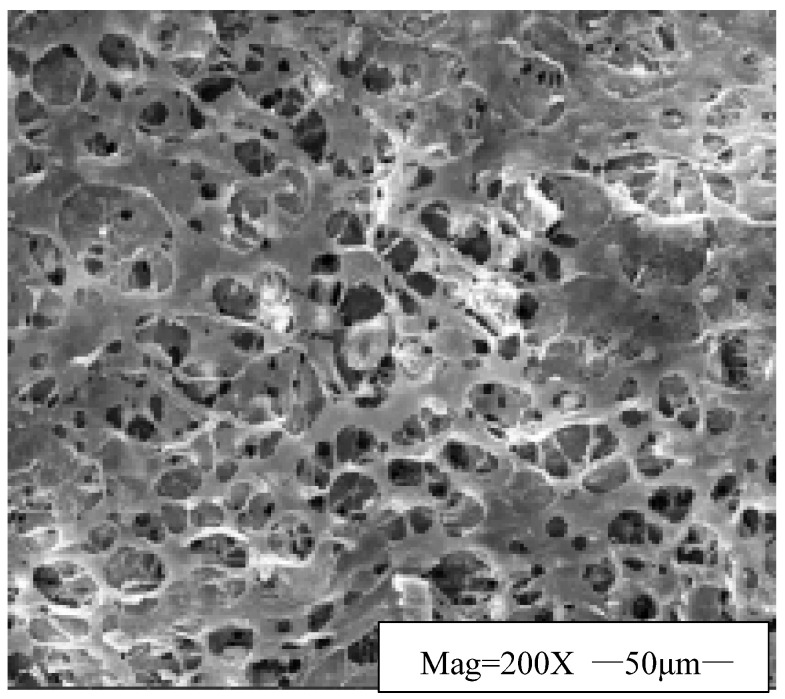
Microstructure of EZFLOW weak gel drilling fluid.

The microstructure of the EZFLOW weak gel drilling fluid was observed using scanning electron microscopy, and the experimental results are shown in Figure 1.

As shown in Figure 1, the microstructure of the EZFLOW weak gel drilling fluid is composed of the network structure which is filled with the whole space and the sol particles embedded in the nodes of the network structure or filled in the interior of the network space, forming a ‘weak gel gelling agent-water-polymer-solid particles’ complex network structure, which is a unique colloidal dispersion system. This unique spatial network gives the EZFLOW weak gel drilling fluid exceptional rheological properties, which manifest in the following ways: (1) the yield point of the EZFLOW weak gel drilling fluid is 15 Pa, and it is relatively high, due to the network’s dense framework and high node density, enhancing sand transport in horizontal sections and wellbore cleaning efficiency; (2) the EZFLOW weak gel drilling fluid has a good shear thinning property, high viscosity (70,000 mPa·s) at low speed (0.3 r/min), a viscosity of 32 mPa·s at high speed (600 r/min), low viscosity at high shear rates which boosts mechanical drilling speed, and high viscosity at low shear rates which suspends drill cuttings; (3) the static gel strength recovery of the EZFLOW weak gel drilling fluid static shear force is not time-dependent. The weak-gel properties quickly prevent cuttings beds in horizontal sections.

#### 2.1.2. Plugging Performance

**Figure 2:**
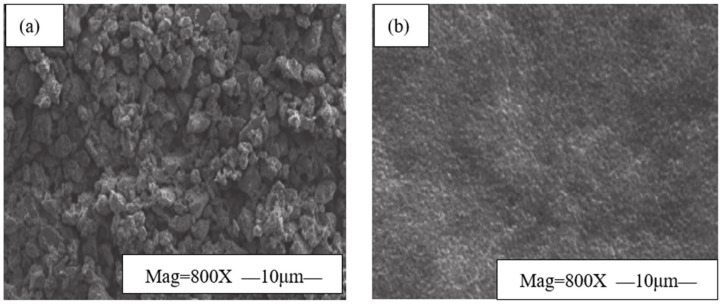
Comparison of sand disk surface morphology before and after PPA test. (**a**) Sand disk surface morphology before the experiment. (**b**) Sand disk surface morphology after the experiment.

A permeability plugging apparatus (PPA) evaluated the plugging effectiveness of EZFLOW weak gel drilling fluid. Under a 3.5 MPa differential pressure, it tested the fluid on a sand disk with a 3 μm pore size. Figure 2 presents the experimental results.

As shown in Figure 2, the sand disk had a random microporous structure before the experiment. After the experiment, its surface pores were filled with dense nanoparticles, indicating effective plugging by the EZFLOW weak gel drilling fluid. This is mainly due to two reasons: (1) EZVIS forms deformable aggregates and films through intermolecular or intramolecular association, rapidly sealing micropores and microfractures; (2) EZCARB, a rigid plugging material, creates physical barriers via bridging mechanisms, increasing the resistance to filtrate invasion.

The dense sealing layer formed by EZFLOW on the sand disk surface enhances the pressure-bearing capacity through the combined physical and chemical effects of EZCARB and EZVIS. This prevents leakage during drilling and completion thanks to the fluid’s dense network framework, high node density, high structural viscosity, and viscoelastic properties.

### 2.2. Construction of Filter Cake Removal Fluid System

The filter cake of EZFLOW weak gel drilling fluid consists mainly of the rheological modifier EZVIS, the fluid loss reducer EZFLO, and the acid-soluble temporary plugging agent EZCARB. The formed mud cake is relatively dense, making it hard to back-produce during later production. Meanwhile, the high-molecular-weight polymer EZVIS can linger in the reservoir, and the invading temporary plugging material EZCARB can block hydrocarbon flow paths. Therefore, during completion, a filter cake removal fluid is needed to eliminate the damage to the reservoir caused by the filter cake, the high-molecular-weight polymer EZVIS, and the temporary plugging material EZCARB, thus enhancing hydrocarbon production.

#### 2.2.1. Acid Selection

Strong acid breakers react rapidly, first with acid-soluble temporary plugging agents like EZCARB, dissolving them 10–20 times faster than the acid-catalyzed hydrolysis of polymers in the filter cake. This often leads to incomplete filter cake removal. Moreover, prolonged contact of strong acids with metal equipment can cause corrosion and pose safety risks. Therefore, a retarded composite organic acid HWCP is chosen for the filter cake removal fluid.

##### Gel-Breaking Performance Evaluation

The molecular chain of the polymer EZVIS degrades into smaller compounds under acid exposure, facilitating the removal of the EZFLOW weak gel drilling fluid filter cake and alleviating reservoir blockage.

(1) Effect of HWCP Acid Solution on EZVIS Solution’s Network Structure

**Figure 3:**
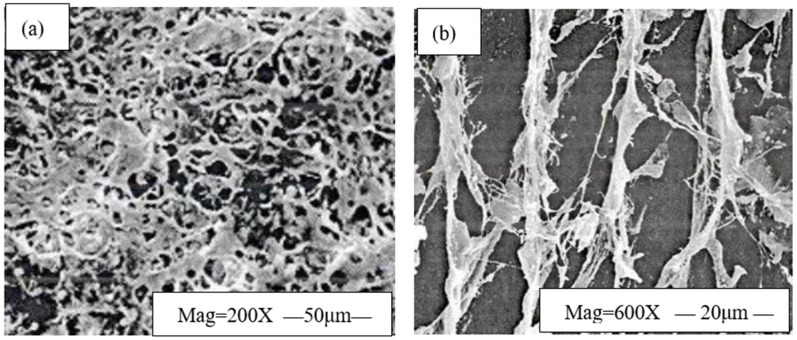
Network structure of 0.8% EZVIS seawater solution before and after treatment with 6% HWCP acid solution. (**a**) Network structure before treatment; (**b**) network structure after treatment.

The network structure of 0.8% EZVIS seawater solution was observed using scanning electron microscopy before and after treatment with 6% HWCP acid solution. The experimental results are shown in Figure 3.

As shown in Figure 3, after treatment with 6% HWCP acid solution, the network structure of 0.8% EZVIS seawater solution was disrupted to some extent, with reduced network density and evident chain scission. The main reasons are as follows: (1) 6% HWCP acid solution disrupts the secondary bonds in the polymer EZVIS that are essential for the formation of its network structure; (2) 6% HWCP acid solution alters the hydration layer of the polymer EZVIS, causing the macromolecular chains to contract and thus damage the network structure; (3) 6% HWCP acid solution causes the molecular chains of the polymer EZVIS to break.

(2) Effect of HWCP Acid Solution Concentration on Apparent Viscosity of EZVIS Solution

**Figure 4:**
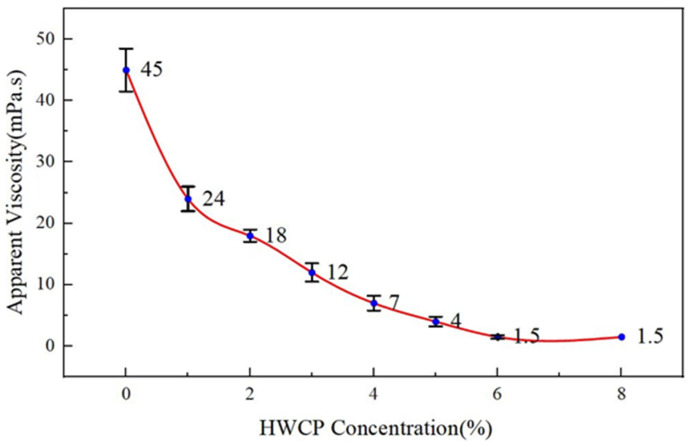
Effect of HWCP acid concentration on the apparent viscosity of 0.8% EZVIS seawater solution.

When the polymer EZVIS degrades, its solution’s apparent viscosity decreases. So, the viscosity change was used to measure the degradation of EZVIS. The results are shown in Figure 4.

Figure 4 shows that the apparent viscosity of 0.8% EZVIS seawater solution decreases with higher HWCP acid concentration. This is because EZVIS degrades into small molecules due to molecular chain scission caused by HWCP acid. HWCP acid has strong gel-breaking ability, which can remove residual EZVIS from the filter cake and reservoir. When HWCP concentration exceeds 6%, the solution’s viscosity approaches that of clean water (1 mPa·s), aiding in the removal of residual liquid. However, too low of an HWCP concentration reduces the degradation efficiency, so 6% HWCP is optimal.

##### Evaluation of Dissolution Capacity

(1) Dissolution Rate of Temporary Plugging Agent EZCARB for HWCP Acid Solution

**Figure 5:**
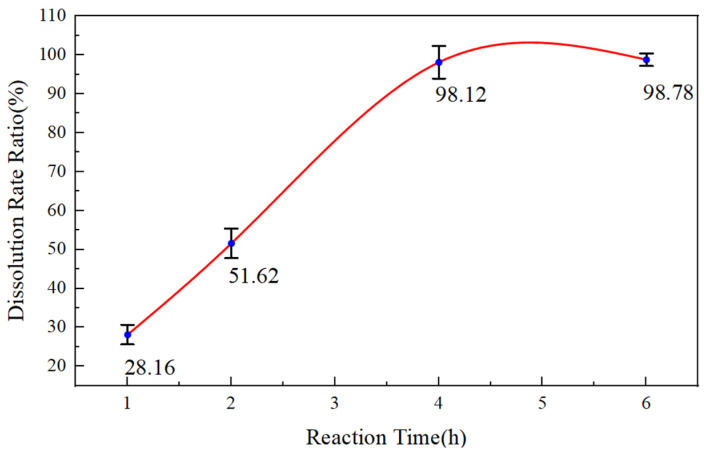
Dissolution rate of temporary plugging agent EZCARB for 6% HWCP acid solution at different reaction times.

The dissolution rate was calculated. The experimental results are shown in Figure 5.

As shown in Figure 5, the dissolution rate of EZCARB for 6% HWCP acid solution increases with reaction time, reaching nearly 100% after 4 h. This indicates that 6% HWCP acid solution has a strong dissolving capacity for EZCARB. The multi-step release of active hydrogen ions by HWCP acid solution contributes to its high dissolution rate and delayed reaction with EZCARB.

(2) Dissolution Rate of Reservoir Cuttings for HWCP Acid Solution

**Figure 6:**
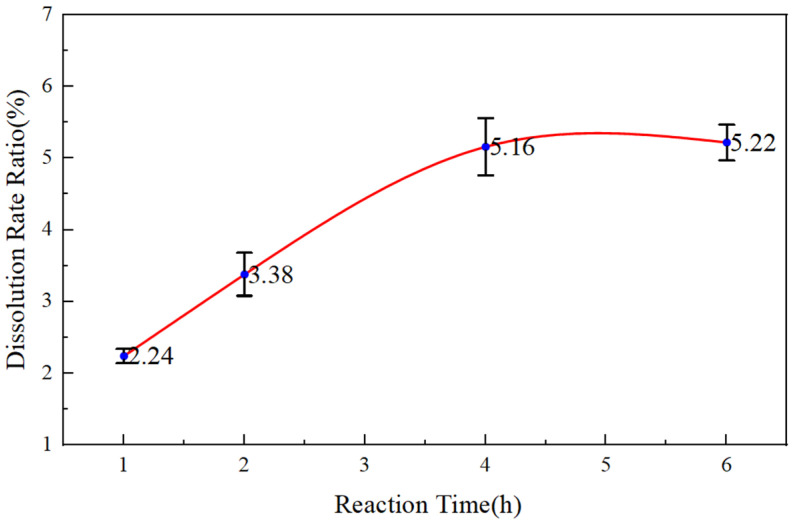
Dissolution rate of reservoir cuttings for 6% HWCP acid solution at different reaction times.

The dissolution rate was calculated. The experimental results are shown in Figure 6.

Figure 6 shows that the dissolution rate of reservoir cuttings for HWCP acid solution increases with reaction time. HWCP acid solution’s multi-step active hydrogen ion release gives it a delayed reaction with the cuttings, and it has a retarding deep-acidizing effect on the reservoir, boosting permeability and production.

(3) Dissolution Rates of EZCARB and Reservoir Cuttings by Different Concentrations of HWCP Acid Solution

The dissolution rates were calculated. The experimental results are shown in Table 1.

Table 1 shows that the dissolution rates of EZCARB and reservoir cuttings increase with the concentration of HWCP acid solution. When the concentration exceeds 5%, the dissolution rate of EZCARB surpasses 90%, and that of reservoir cuttings exceeds 5%. However, too low a concentration of HWCP acid leads to incomplete removal of the filter cake, while too high a concentration can cause excessive dissolution of the reservoir rocks, damaging the reservoir’s framework and causing secondary damage. Therefore, the optimal concentration of HWCP acid solution is 6%.

#### 2.2.2. Dosage Optimization of Corrosion Inhibitor HWCI

The acid solution HWCP exhibits certain corrosivity to metals, which can lead to significant corrosion damage to field equipment. This not only incurs additional costs but may also pose safety risks under severe conditions. Therefore, a specific amount of corrosion inhibitor must be incorporated into the filter cake removal fluid to mitigate its corrosion rate. The experimental results are summarized in Table 2.

As shown in Table 2, adding the corrosion inhibitor HWCI to the 6% HWCP acid solution effectively reduces the corrosion rate. This is because the HWCI molecules, containing nitrogen and oxygen atoms with unshared electron pairs, form positively charged cations in the acid solution. These cations absorb onto the metal surface via electrostatic attraction and van der Waals forces, creating a protective monomolecular adsorption film. This film prevents H^+^ ions from reaching the metal surface and inhibits the cathodic process of hydrogen ion reduction. The corrosion rate decreases and the inhibition efficiency increases with higher doses of HWCI. However, when the HWCI dosage exceeds 3%, changes in corrosion rate and inhibition efficiency become minimal. Therefore, the optimal dosage of HWCI is 3%.

### 2.3. Performance Evaluation of Filter Cake Removal Fluid System

The filter cake removal fluid for EZFLOW weak gel drilling fluid is composed of filtered seawater, 6% composite organic acid HWCP, and 3% corrosion inhibitor HWCI. Its effectiveness in removing the filter cake and protecting the reservoir was assessed.

#### 2.3.1. Evaluation of Filter Cake Removal Ability

**Figure 7:**
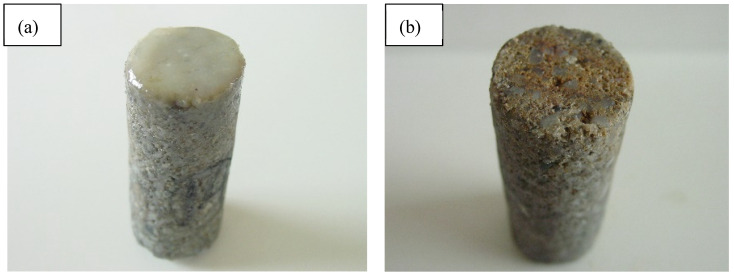
Morphology of core contamination end before and after treatment with filter cake removal fluid. (**a**) Core contamination end before soaking in filter cake removal fluid; (**b**) core contamination end after soaking in filter cake removal fluid.

The experimental results are presented in Figure 7.

As shown in Figure 7, under specific temperature and pressure conditions, the EZFLOW weak gel drilling fluid forms a filter cake on the surface of the reservoir core. However, after treatment with the filter cake removal fluid, the filter cake at the core end face is thoroughly eliminated. This phenomenon is primarily attributed to the following mechanisms: (1) The composite organic acid HWCP in the removal fluid chemically dissolves the temporary bridging agent EZCARB embedded within the filter cake. This dissolution destabilizes the cake’s structural framework, leading to its loosening and subsequent detachment; (2) simultaneously, the high-molecular-weight polymer EZVIS undergoes chain scission under the action of HWCP, degrading into low-molecular-weight compounds. This reduction in polymer molecular weight weakens the cohesive forces binding the filter cake to the core surface, further facilitating its removal.

#### 2.3.2. Reservoir Protection Performance Evaluation

**Figure 8:**
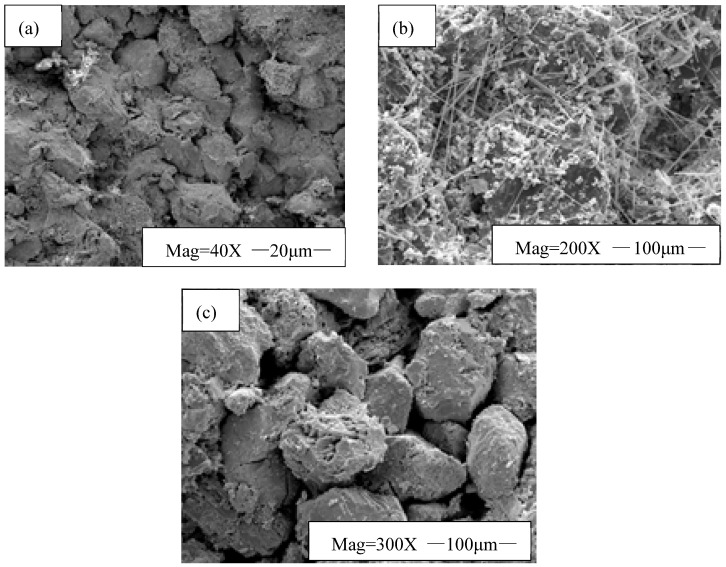
Initial and treated core end faces under different contamination media. (**a**) Initial core end face; (**b**) core end face after drilling fluid contamination; (**c**) core end face after filter cake removal fluid treatment.

Reservoir cores of the Western South China Sea oilfield were used as experimental subjects. The formation damage caused by EZFLOW weak gel drilling fluid and the reservoir protection performance of the filter cake removal fluid were evaluated. The experimental results are shown in Table 3. Scanning electron microscopy was used to analyze the core end faces before and after treatment with the filter cake removal fluid, and the results are presented in Figure 8.

As shown in Table 3, after treatment with the filter cake removal fluid, the permeability recovery of reservoir cores contaminated by EZFLOW weak gel drilling fluid exceeds 95%. This is mainly because the filter cake removal fluid removes the filter cake from the core end face, clears seepage channels, and the composite organic acid HWCP in it provides a deep-acidizing effect, enhancing reservoir permeability. As shown in Figure 8, the initial end-face scanning electron microscopy (SEM) analysis of the core reveals clearly visible pores and throats. After contamination by the EZFLOW weak gel drilling fluid, filamentous and agglomerated polymer EZVIS blocked the core pore throats. However, following treatment with the filter cake removal fluid, the molecular chains of polymer EZVIS fractured and degraded into small-molecule compounds, restoring the core pore throats to their initial state. Meanwhile, the filter cake removal fluid dissolved the temporary plugging agent EZCARB in the filter cake, disrupting its structure. The loosened filter cake subsequently detached, leading to the recovery of the core’s permeability. In conclusion, this filter cake removal fluid demonstrates robust reservoir protection capabilities.

## 3. Conclusions

(1) The microstructure of EZFLOW weak gel drilling fluid consists of a reversible network and sol particles either embedded within the network or encapsulated at its nodes. This unique spatial network endows the system with exceptional rheological properties, good plugging performance, and lubricity.
(2) Based on the components of EZFLOW weak gel drilling fluid’s filter cake, a filter cake removal fluid formula was established: filtered seawater + 6% composite organic acid HWCP + 3% corrosion inhibitor HWCI. This fluid not only removes the filter cake, degrades the polymer EZVIS, and dissolves the temporary plugging agent EZCARB to protect the reservoir, but also provides a retarded deep-acidizing effect, enhancing reservoir permeability and productivity.
(3) After treatment with the filter cake removal fluid, the filter cake on the core end face of the EZFLOW weak gel drilling fluid-contaminated reservoir core is almost completely removed, with a core permeability recovery rate of over 95%. This indicates the fluid’s excellent filter cake removal and strong reservoir protection capabilities.

## 4. Materials and Methods

### 4.1. Materials

EZVIS, a key rheological modifier made from biopolymers and natural polymers, forms high-viscosity weak gels at low shear rates. EZFLO is a modified starch-based fluid loss reducer, and EZCARB is an acid-soluble temporary plugging agent mainly composed of calcium carbonate. JLX serves as a polyalcohol-based shale inhibitor and lubricant. HWCP is a mixture of weak polyacids and organic acid salts. Its ionization happens in five steps: (1) H_5_R → H^+^ + H_4_R^−^, pK_1_ = 2.0; (2) H_4_R^−^ → H^+^ + H_3_R^2−^, pK_2_ = 2.5; (3) H_3_R^2−^ → H^+^ + H_2_R^3−^, pK_3_ = 5.0; (4) H_2_R^3−^ → H^+^ + HR^4−^, pK_4_ = 10.4; (5) HR^4−^ → H^+^ + R^5−^, pK_5_ = 12.0. Under reservoir temperature conditions, HWCP ionizes to release H^+^, which dissolves EZCARB and generates multiple carboxylates to chelate metal ions, preventing secondary precipitation. The corrosion inhibitor HWCI is formulated by blending trans-cinnamaldehyde, alkynol, organic amine dispersant, thiourea, and imidazoline, with the following composition: 62.6% water + 3.4% trans-cinnamaldehyde + 4% alkynol + 5% thiourea + 15%imidazoline. The core sample is the gravelly coarse sandstone in the reservoir section of the Western South China Sea, and the air permeability is 1000 m darcy.

### 4.2. Methods

The experimental method for evaluating how the HWCP acid concentration affects the apparent viscosity of EZVIS solution was as follows: after adding 1%, 2%, 3%, 4%, 5%, 6%, and 8% HWCP acid solution to 0.8% EZVIS seawater solution, stirring evenly, and reacting at 90 °C for 4 h, the apparent viscosity was tested at room temperature. The apparent viscosity measurement steps are as follows: (1) the EZVIS aqueous solution is fully stirred, and then poured into the measuring cup so that the liquid level is in line with the scale line of the viscometer outer cylinder. (2) Set the viscometer speed at 600 r/min and read the data after the dial is stable. (3) The apparent viscosity calculation formula is 1/2 × Φ600; here, Φ600 represents the 600 r/min reading value of the direct-reading viscometer.

The experimental method for evaluating the dissolution rate of EZCARB temporary plugging agent in HWCP acid solution was as follows: A total of 5.0 g of the temporary plugging agent EZCARB was weighed and added to 100 mL of 6% HWCP acid solution at a solid–liquid mass–volume ratio of 1:20. The mixture was reacted at 90 °C for 1, 2, 3, and 4 h, respectively. The experimental method for evaluating the dissolution rate of reservoir cuttings for HWCP acid solution was as follows: A total of 5.0 g of reservoir cuttings from the Western South China Sea oilfield, retained on a 100-mesh sieve, was mixed with 100 mL of 6% HWCP acid solution at a solid–liquid mass–volume ratio of 1:20. The mixture was reacted at 90 °C for 1, 2, 3, and 4 h, respectively.

The experimental method for evaluating the dissolution rate of EZCARB and reservoir cuttings for different concentrations of HWCP acid solution was as follows: A total of 5.0 g of temporary plugging agent EZCARB and 5.0 g of reservoir cuttings (retained on a 100-mesh sieve) were respectively mixed with 100 mL of HWCP acid solution at concentrations of 1%, 3%, 5%, 6%, and 8%, at a solid–liquid mass–volume ratio of 1:20. The mixture was reacted at 90 °C for 4 h, respectively.

The experimental method for optimization of corrosion inhibitor HWCI dosage for N80 steel coupons involved them being immersed in a 6% HWCP acid solution containing different doses of HWCI, followed by subjecting the system to static immersion at 90 °C for 4 h, and the corrosion inhibition performance was assessed using the static coupon weight loss method.

The experimental method for the evaluation of filter cake removal ability was as follows: using a high-temperature and high-pressure core dynamic damage experimental device, the EZFLOW weak gel drilling fluid contaminated the core under dynamic conditions. The experimental conditions were as follows: a temperature of 90 °C, a pressure difference of 3.5 MPa, a velocity gradient of 300 s^−1^, and a contamination time of 125 min. Subsequently, the contaminated core ends were soaked in the filter cake removal liquid at 90 °C for 4 h.

The experimental method for the evaluation of reservoir protection performance was as follows: according to the SY/T6540-2021 “Laboratory Evaluation Method of Drilling and Completion Fluid Damage to Oil Formations”, the following experimental steps were undertaken: (1) The core of the Western South China Sea oilfield reservoir is saturated with formation water, and the core permeability K_1_ is determined by forward displacement with white oil; (2) under the conditions of a pressure difference of 3.5 MPa, temperature of 90 °C, shear rate of 300 s^−1^, and contamination time of 120 min, the weak gel drilling fluid is reversely contaminated to the core; (3) the permeability K_2_ of the core after the contamination of the weak gel drilling fluid is determined by forward displacement, and the permeability recovery value K_2_/K_1_ × 100% is calculated; (4) the filter cake removal fluid with 2 times pore volume is injected reversely into the contaminated core, and it is placed at 90 °C for 4 h; (5) the permeability K3 of the core after the treatment of the filter cake removal fluid is determined by forward displacement, and the permeability recovery value K_3_/K_1_ × 100% was calculated.

## Figures and Tables

**Table 1:** Dissolution rates of EZCARB and reservoir cuttings by different HWCP acid concentrations.

HWCP Concentration (%)	EZCARB Dissolution Rate (%)	Dissolution Rate of Reservoir Cuttings (%)
1	70.02	2.32
3	84.86	4.52
5	96.86	5.02
6	98.12	5.16
8	99.28	5.48

**Table 2:** Corrosion rates and inhibition efficiencies of HWCI at different dosages.

Solution	HWCI Concentration (%)	Corrosion Rate (g/m²·h)	Inhibition Efficiency (%)
Filtered seawater + 6% HWCP	0	1.204	--
	1.0	0.371	69.19
	2.0	0.103	91.45
	3.0	0.052	95.68
	4.0	0.049	95.93
	5.0	0.045	96.26

**Table 3:** Experimental results of reservoir protection performance of filter cake removal fluid.

Contamination Media	K_1_ (mD)	K_2_ (mD)	K_3_ (mD)	K_2_/K_1_ (%)	K_3_/K_1_ (%)
EZFLOW weak gel drilling fluid	6.763	5.137	--	75.96	---
EZFLOW weak gel drilling fluid + filter cake removal fluid	8.471	6.526	8.189	77.04	96.67

K_1_ is the original permeability of the core, K_2_ is the permeability of the core contaminated by EZFLOW weak gel drilling fluid, K_3_ is the permeability of the core after being contaminated by EZFLOW weak gel drilling fluid and then treated by filter cake removal fluid.

## Data Availability

The data presented in this study are openly available in the article.
